# Enhanced strength of ultrasonically-welded austenitic stainless steels joints by introducing dynamic recrystallization of interlayers

**DOI:** 10.1038/s41598-024-66205-8

**Published:** 2024-08-02

**Authors:** Yun-Ta Chung, Hue-En Chu, Yu-Hsuan Juan, Yo-Lun Yang, Jhe-Yu Lin

**Affiliations:** 1 Department of Mechanical Engineering, National Taipei University of Technology, No. 1, Sec. 3, Zhongxiao E. Rd., Taipei, 10608 Taiwan; 2 Graduate Institute of Manufacturing Technology, National Taipei University of Technology, No. 1, Sec. 3, Zhongxiao E. Rd., Taipei, 10608 Taiwan

**Keywords:** Ultrasonic welding, Stainless steels, Interlayer, Dynamic recrystallization, Materials science, Structural materials

## Abstract

This study investigated the role of interfacial deformability in bond integrity and strength, particularly in the production of robust joints between harder austenitic stainless steels (SS) during ultrasonic welding. The specimen without the interlayer experienced limited strength enhancement owing to internal cracking from continuous sliding at interfacial temperatures below 0.6 times the melting point (T_m_), which is attributed to the limited deformability of the austenitic SS. In contrast, introducing Fe and Ni interlayers between the substrates resulted in a notable increase in the interfacial strength, surpassing 2500 N in the peak load within a reduced welding duration. The correlation between the interfacial strength and the peak temperature suggests that a substantial decrease in hardness below 0.4 T_m_ is sufficient for extensive bond formation. Moreover, dynamic recrystallization (DRX) led to grain refinement in the Fe interlayer owing to shorter weld durations, whereas grain growth was observed in the Ni interlayer due to higher peak temperatures. Both the Fe and Ni interlayers significantly improved the bonding integrity by accommodating plasticity through the above phenomena without severe damage to the substrates, leading to increase of interfacial strength by 24% (2050 N to 2500 N) and reduction of weld duration by 40% (1.5 s in Fe interlayer). In addition, the fracture position after the lap shear test shifted from the edge of the weld area to the SS substrate.

## Introduction

Ultrasonic welding (USW) is considered an energy-saving assembly process due to its low bonding temperature, short weld duration, and fewer workpiece microstructural changes^1^. During USW, rapid heat generation and deformation from sliding friction are produced to form a weld, and the bonding mechanisms include severe plastic deformation (SPD) with the occurrence of dynamic recrystallization (DRX), followed by bond achievement and the disappearance of internal defects^2,3^. DRX has been recognized in thermomechanical processes, particularly in solid-state processing at elevated temperatures, such as hot deformation and friction stir welding^4^. Under DRX, grain refinement^5^ and removal of the existing plastic strain^6^ have been reported, resulting in enhanced deformability at the interface. For example, in the USW of Al, Cu, or Mg alloys, rapid sliding at the interface causes concentrated plastic strain and temperature increase, producing a gradient distribution of the microstructure with sufficient interfacial strength^7–13^, which is a result of DRX and plastic yielding at elevated temperatures.

Meanwhile, USW of metals with higher melting points can be more challenging owing to their resistance to plastic deformation at elevated temperatures^14^. It has been reported that high-alloyed steels exhibit a slight decrease in hot hardness compared to pure metals^15^. In addition, grain refinement increases hardness with decreasing ductility, which poses risks for fractures formed locally during sliding friction^16^. Austenitic steels subjected to rapid sliding undergo surface deformation and grain refinement. This grain refinement strengthens the surface and enhances the shear deformation resistance, lowering the plastic strain^17^.

Further, interlayers are feasible to accommodate concentrated plastic strain, enabling bonding mainly via improved contact to reduce interfacial defects, quick temperature rises, and localized plastic deformation^18^. For example, an Al interlayer is utilized in welding harder metals such as steel/Ti owing to its ductility and capability to induce large plastic deformation at the interface^19^. In contrast, a Ni interlayer requires high temperatures and extended welding duration to allow diffusion into Ti and convert α-Ti to the more deformable β-Ti phase as the interfacial temperature reaches above β-transus point (765 °C) to facilitate bonding strength evolution^19^. Recent studies also unveil the influence of interfacial temperature on USW of α-Ti sheets with Fe interlayer by rising temperature above β-transus point to create a heterogeneous α/β/α interface, significantly improving interfacial strength^20^ and demonstrating the impact of deformability and peak temperature while using interlayer in USW.

These studies suggested that deformability at elevated temperatures with SPD could play a significant role in bond development, but the intrinsic hardness of the substrates could inhibit that bond formation with DRX. For instance, austenitic steels are less prone to forming integrated bonds because of their relatively high yield strengths, even at high temperatures^21^. This can be improved by introducing interlayers to induce greater plastic strain through a heterogeneous interface design. Pure Fe and Ni were employed as interlayers because of their excellent compatibility (no reactivity with steels^22,23^) and solubility in austenitic steels to compensate for the excessive plastic strain that occurs during USW. Thus, this study aims to clarify the influence of deformability near the interface at elevated temperatures and its effect on joint integrity.

## Experimental methods

316L stainless steel (SS) sheets (Fe-17.2Cr-12.1Ni-1.5Mo-0.02C in wt.%) with dimensions of 20 × 10 × 0.5 mm were used as specimens. Before joining, the 316L was annealed at 1080 °C for 2 min, followed by water-quenching to obtain a fully recrystallized austenite microstructure. After annealing, the ultimate tensile strength was 485 MPa. Fe and Ni foils with a thickness of 10 µm were used as interlayer metals. Figures S1-S2 of the Supplementary Information and Table 1 show their microstructures before USW and hardness values. Before joining, the 316L sheets were mirror-polished to obtain a smooth surface without contaminants or oxides. Previous studies have shown that smooth surfaces increase rapidly by facilitating larger contact areas^24^.

**Table 1:** Hardness of 316L SS, Fe, and Ni foils.

Metals	Hardness (HV_0.05_)
316L SS substrate	156
Fe foils	84
Ni foils	144

**Figure 1:**
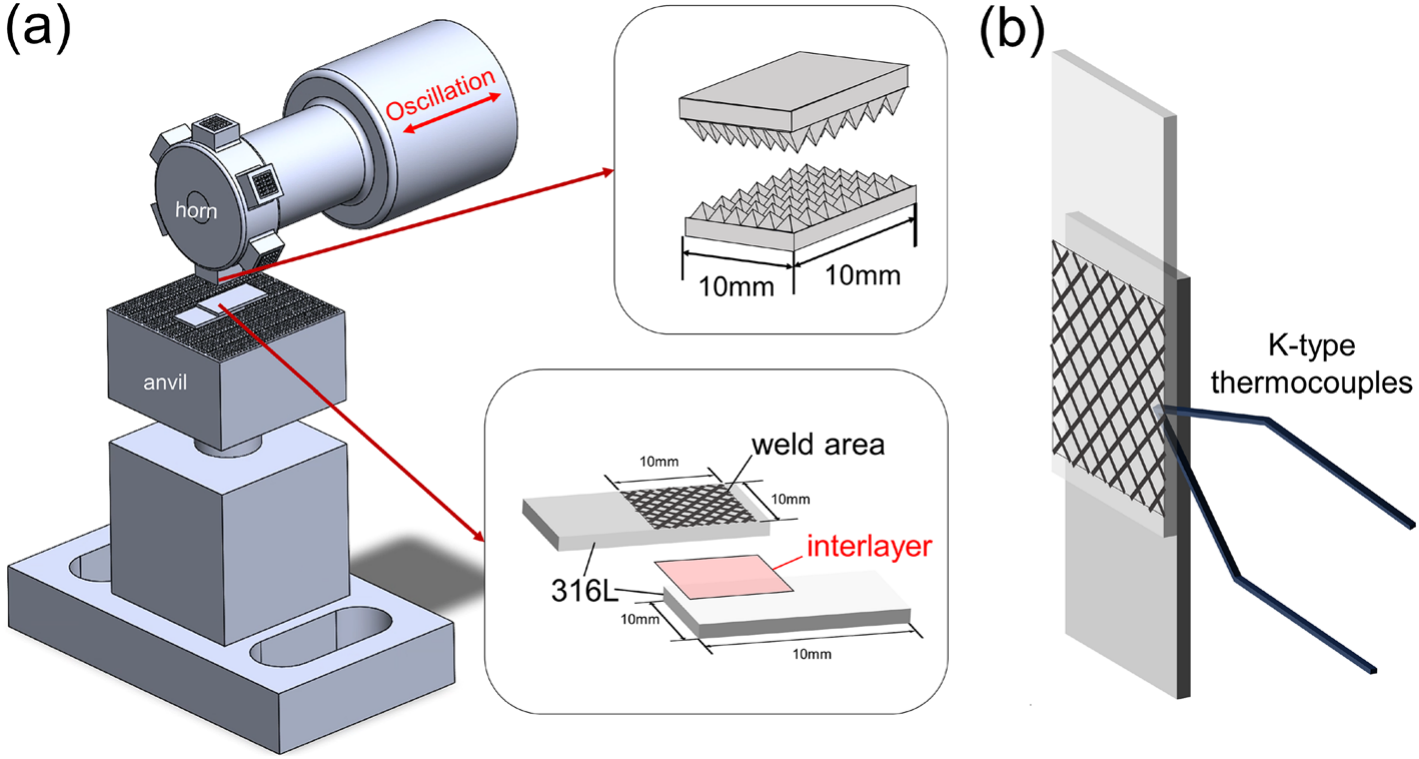
Schematics of (**a**) setup of USW and welding specimen (**b**) temperature measurement using K-type thermocouples.

The USW setup to join two specimens is shown in Fig. 1a using an ultrasonic metal welder (SG-1580 ultrasonic metal welder, Telsonic Inc.); the welding frequency was 15 kHz, the oscillation amplitude was 35 µm, the clamping force was 1500 N and horn dimensions were 10 × 10 mm. During the USW, K-type thermocouples (0.127 mm in diameter) were placed at the welding area's edge to measure the specimen's surface temperature (Fig. 1b). After joining via USW, a lap shear test was conducted using universal testing equipment (TPP-2000, Pingtai Inc.) to evaluate the interfacial strength.

In addition to the lap shear test, a microstructural analysis was performed using a cross-sectional polisher (Ion Milling System IM4000 Plus, Hitachi; CP) to obtain a clean cross-section. After surface preparation, microstructural analyses were conducted using a scanning electron microscope (SEM, SU-5000, Hitachi) equipped with energy dispersive spectroscopy (EDS) and electron backscattered diffraction (EBSD, Oxford Instruments Inc.) for interfacial microstructure observation and crystallographic analysis.

## Results

### Evolutions of interfacial strength

**Figure 2:**
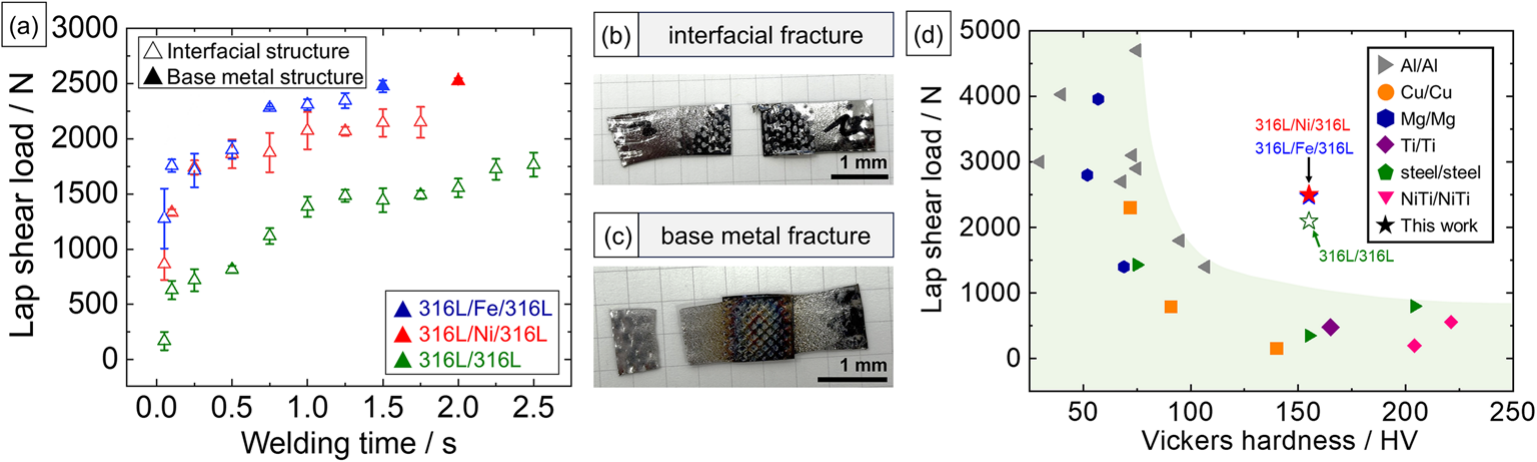
(**a**) Strength-time relationship of the joints using different interlayers, appearances of (**b**) interfacial fracture, (**c**) base metal fracture, and (**d**) comparison between lap shear load and hardness of base metals with previous works.

Figure  2a shows the relationship between the lap shear strength and welding time. In the 316L/316L interface without interlayer, interfacial strength increased slowly while welding time increased to 2.5 s with 2000 N. Regarding lap shear test results for this welding time, a fracture occurred at the edge of the weld area, which could be attributed to thinning of the substrate due to stress concentration at the edge of the weld area. Without an interlayer, the maximum strength achieved was 80% of the substrate strength (approximately 2500 N).

Conversely, adding interlayer metals (denoted as 316L/Fe/316L and 316L/Ni/316L interfaces) significantly enhanced interfacial strength. In two instances, this resulted in an increase in interfacial strength from approximately 2000–2500 N with a welding time of less than 2.0 s. Substrate fracture occurred after 1.5 s welding time for the 316L/Fe/316L interface and after 2.0 s for the 316L/Ni/316L interface. Both cases exhibited a peak lap shear load of approximately 2500 N. The Fe and Ni interlayers exhibited interfacial strengths of more than 1000 N after 0.1 s welding. In detail, the 316L/Fe/316L interface gradually increased to 2000–2400 N in welding times ranging from 0.75–1.25 s and peaked at 2500 N after 1.5 s of welding. Similarly, the 316L/Ni/316L interface revealed a saturated increase of interfacial strength after 0.25 s of welding, but peak interfacial strength was delayed after 2.0 s of welding. The difference in the evolution of strength corresponds to the difference in hardness. In other words, softer interlayer metals exhibited a pronounced effect in accelerating the evolution of strength to its peak strength.

Figure 2b and c present the appearances of the welded specimens after the lap shear tests. A fracture occurred at the bonding interface because the interfacial strength was lower than that of the 316L substrate. At a long weld duration, the interfacial strength of the joints was equal to or higher than that of 316L, resulting in the fracture of the 316L substrate. Considering the cross-Sect. (5 mm^2^) perpendicular to the loading direction, this peak load value corresponded to the ultimate tensile strength of the 316L substrate (ultimate tensile strength (UTS) of 485–515 MPa^25^ applied vertically on a cross-sectional area of 10 × 0.5 mm; 2425–2575 N lap shear load), suggesting improved bonding without damaging the substrate. A previous study reported that the interlayer increased the joining strength and reduced the weld duration required to obtain a high strength^20^.

Figure 2d compares the lap shear loads and base metal hardness across the USW of similar metals welded with different weld area sizes (e.g., Cu/Cu^26–28^, Al/Al^7,29–35^, Fe/Fe^22,36^, Mg/Mg^10,37,38^, NiTi/NiTi^39,40^, and Ti/Ti^41^). The comparison shows that the lap shear load tends to decrease as the hardness of the base metal increases, thereby highlighting the impact of metal hardness on joint robustness. Conversely, our results revealed an enhanced strength at a comparatively higher hardness for the base metals. This can be attributed to the interlayer utilization on 316L substrates, which has a moderate base metal hardness.

### Bonding Development of the 316L/316L interface without Interlayers

**Figure 3:**
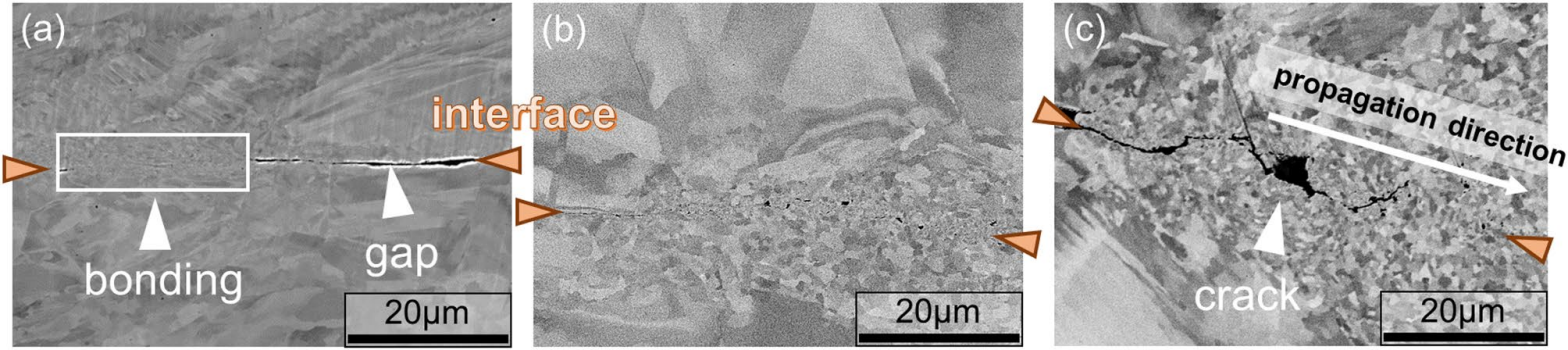
Microstructure of bonding interface after (**a**) 0.25 s, (**b**) 2.0 s, and **c** () 2.5 s of welding.

Fig. 3 illustrates the development of the interfacial microstructure during different stages of welding. Fig. 3a shows the band-like distribution of bonds formed at the interface after 0.25 s welding time (white arrows), where plastic deformation occurred under sliding with heavy loading. When the welding time is prolonged to 2.0 s, an ultrafine-grained structure without gaps becomes evident, as shown in Fig. 3b. Fig. 3c shows that after 2.5 s welding, cracks gradually appeared and propagated horizontally at the joint interface (white arrows).

Previous studies have reported that grain refinement after severe and local sliding deformations can improve wear resistance through surface work hardening and grain boundary strengthening effects^42,43^. However, the formation of ultrafine grains under a higher degree of plastic strain during USW can lead to a decrease in toughness and the propagation of internal cracks, thereby causing fractures adjacent to the interface^44,45^. Because bonding development is a competition between bonding formation from local deformation and bonding destruction under USW sliding^46^, these phenomena can explain the saturated strength evolution when the welding time increases over 2.0 s at the 316L/316L interface, as shown in Fig. 2.

During the early stages of USW, both bonding surfaces produce severe plastic deformation (SPD) sections under high-frequency oscillations and heavy loads. This caused heavy sliding between the metals and produced ultrafine grains at the welding interface. As the welding time increases, the ultrafine-grained regions expand further away from the interface. However, cracks propagated and became noticeable as welding time was prolonged to more than 2.0 s. This can be attributed to the higher resistance of austenite nanograins to plastic deformation. As reported in previous studies, Fe–Cr-Ni stainless steels exhibit an insignificant drop in hardness (approximately 50%) in the temperature range of 200–700 °C^47^, which is lower than those in pure Fe or Ni (showing a hardness drop of 80–90% from 600–800 °C)^48^. With grain refinement near the interface and relatively high hardness, it is suggested that the 316L/316L interface possesses a lower capacity for deformability at elevated temperatures, leading to crack formation in the bonded areas. Interlayers were used to prevent crack formation at the bonding section of the 316L/316L interface.

### Bonding development of the 316L/Fe/316L Interface

**Figure 4:**
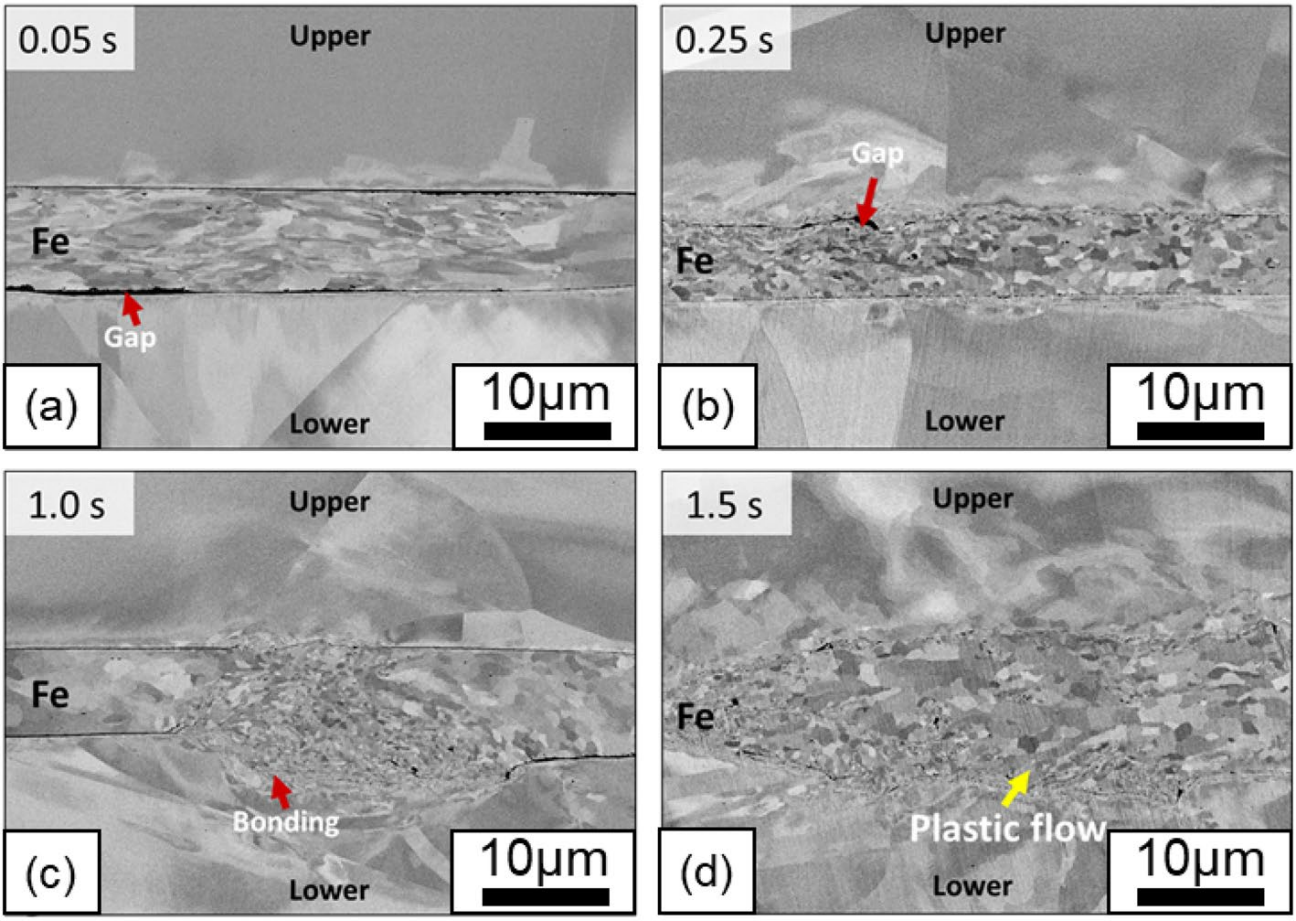
Interfacial microstructures of 316L/Fe/316L interface after (a) 0.05 s, (b) 0.25 s, (c) 1.0 s, and (**d**) 1.5 s of welding.

Figure 4 shows the time-dependent evolution of the microstructure at the 316L/Fe/316L interface. Compared with the 316L/316L interface, the SEM image revealed a reduction in the gaps in both the upper 316L/Fe and Fe/lower 316L gaps as the welding time increased. As shown in Fig. 4a (0.05 s), partial bonding was achieved at the upper 316L/Fe interface, whereas noticeable dark gaps remained at the lower Fe/316L interface, suggesting a lower strength (approximately 1300 N, as shown in Fig. 2). As the welding time increased to 0.25 s (Fig. 4b), the plastic flow became evident (indicated by arrows) at the observed wavy interface, suggesting that local deformation gradually developed. After 1.0 s of welding, Fig. 4c shows a significant gradual increase in the bonding area at the lower Fe/316L interface. The middle region, which appears thicker in the SEM image, indicates that 316L was subjected to local plastic deformation owing to intermixing of the Fe interlayer. Regarding the similar contrast shown in the backscattered SEM image, the SEM images overlaid with the EDS maps and line profiles shown in Figure S3 in the supplementary information indicate where the bonding interface was formed with the introduction of the interlayers.

After 1.5 s of welding, the Fe foil was intermixed with the upper and lower 316L substrates, allowing the formation of direct contact between the substrates to create bonded sections, as shown in \* MERGEFORMAT Fig. 4d. Only minor voids remained, and the gaps at the 316L/Fe and Fe/316L interfaces almost disappeared. The backscattered contrast in the SEM image suggests grain refinement in the Fe interlayers. In addition, the thickness of the Fe interlayers was reduced less owing to their higher hardness compared with the Al interlayer used in previous studies^19^. A fine-grained section in the Fe interlayer appeared and increased owing to SPD, followed by an increase in temperature. This may be closely related to the occurrence of DRX, an interfacial phenomenon in the bond development of USW^2^.

**Figure 5:**
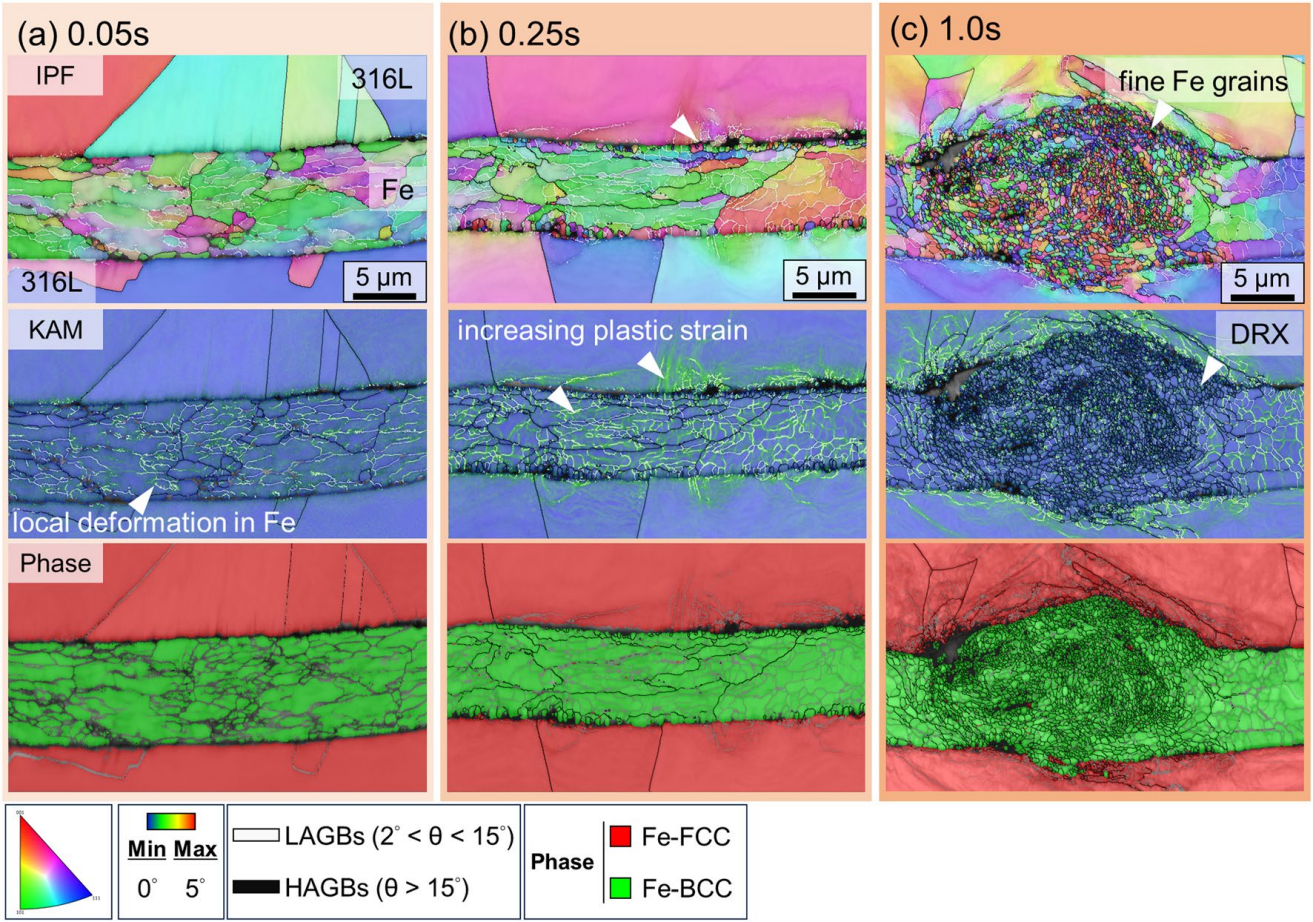
Time-dependent interfacial microstructures of the 316L/Fe/316L interface after (a) 0.05 s, (b) 0.25 s, and (c) 1.0 s of welding, characterized by EBSD.

Figure 5 shows the IPF map of the 316L/Fe/316L interface. The IPF map in Fig. 5a (0.05 s) shows that the Fe grains are deformed with a rolled microstructure in the Fe interlayer during the early stages of USW. The average grain size under these conditions is 4.7 µm. The KAM map in Fig. 5a suggests the presence of localized plastic strain in the deformed grains, whereas the 316L grains near the interface do not exhibit significant strain. This microstructure indicates that plastic deformation was more predominant in the Fe interlayer than in the 316L substrate. Phase maps provide a more explicit expression for distinguishing the bonding interface, where green denotes BCC-Fe and red denotes FCC-Fe. After 0.25 s of welding Fig. 5b), grains in the Fe interlayer stretched along the horizontal direction with a more pronounced formation of low-angle grain boundaries (LAGBs), as shown in the IPF map. The KAM map also indicated an increase in the plastic strain in the Fe interlayer, corresponding to the LAGBs shown in the IPF map. In addition, the residual stress increased on the 316L side near the interface, implying a greater extent of plastic deformation at the upper 316L/Fe and Fe/lower 316L interfaces.

As the welding time increased to 1.0 s (Fig. 5c), the fine-grained region became rounded and more compact, similar to the wear particle formation reported in previous studies^11,23^. The IPF maps show a thickened area of fine Fe grains with significant deformation in the 316L substrate, whereas the KAM map demonstrates a noticeable reduction in the plastic strain in the Fe interlayer. Additionally, the LAGBs in the Fe interlayer progressively disappeared and were replaced by dense high-angle grain boundaries (HAGBs), forming a highly fine-grained microstructure with plastic flow. This microstructural evolution was similar to that observed in the specimens without an interlayer.

**Figure 6:**
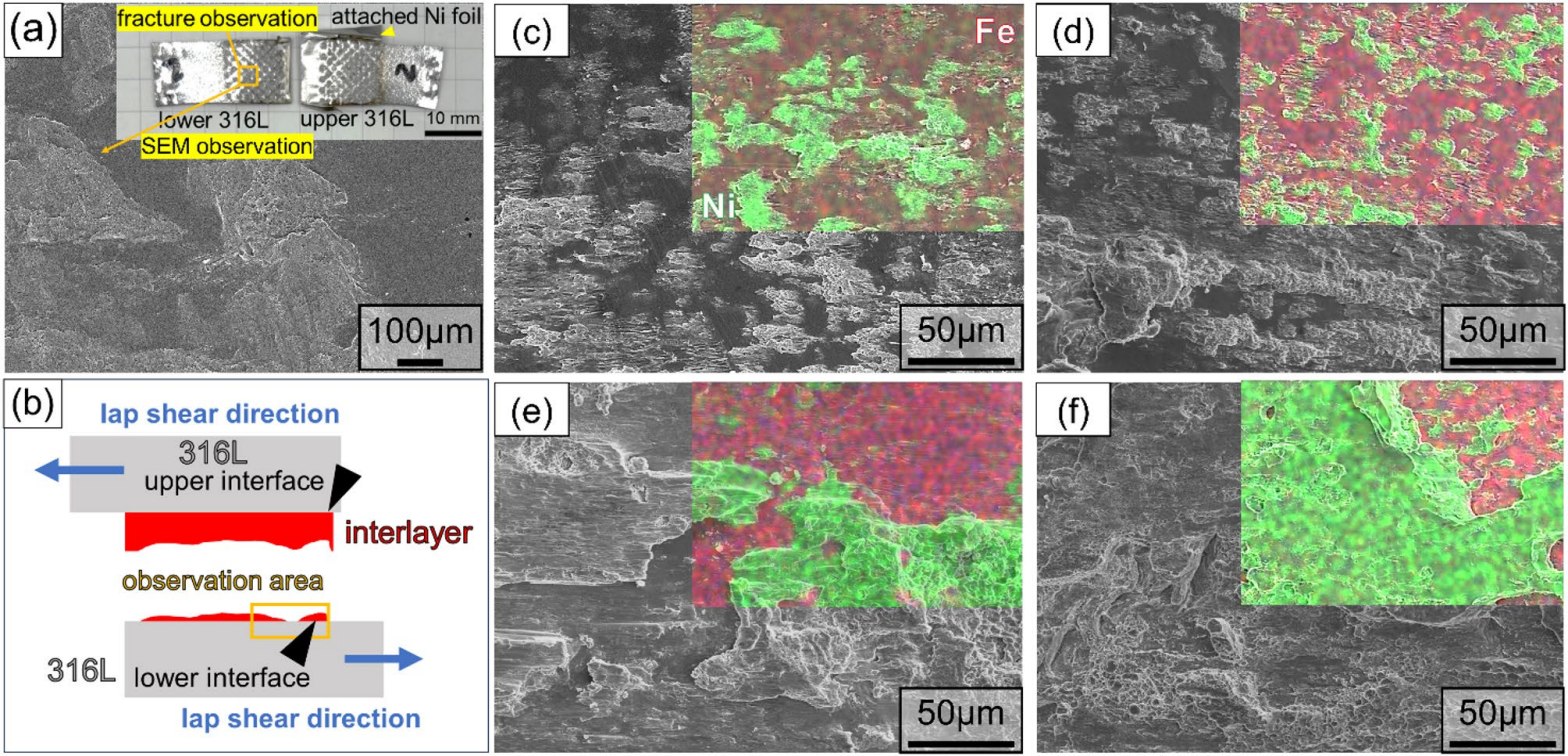
(**a**) SEM image of fractured surface with interfacial fracture (**b**) schematic of observation position at fractured surface, and fractured surfaces with Fe interlayers after (**a**) 0.25 s, (**b**) 0.5 s, (**c**) 1.0 s, and (**d**) 1.25 s welding, characterized by SEM and EDS.

Fig. 6a shows the fractured surfaces of the 316L/Fe/316L interface corresponding to the interfacial microstructure. The fracture observations were conducted at the lower Fe/316L interface because fractures predominantly occurred within the Fe or Ni foils near the lower Fe/316L interface with the subsequent attachment of metal fragments to the bottom 316L substrate (Fig. 6b). Comparatively, no attachment of 316L fragments was found on the interlayer side, which is attributable to the higher toughness of 316L compared to pure Ni or Fe. Previous studies^19^ also observed crack propagation within a relatively soft interlayer near the bottom substrate/interlayer interface.

After 0.25 s of welding (Fig. 6c), the overlaid image highlights the attachment of Fe to the 316L side, denoted in green for Fe and red for Ni. This attachment of the Fe interlayer has been reported in previous studies in which fractures occurred in metals with lower hardness and strength. After 0.5 s of welding (Fig. 6d), the fraction of attached Fe increased to a larger size, suggesting bonding development with prolonged welding duration. In addition, an increase in the Fe attachment was observed as the welding time increased (Fig. 6e,f).

This trend suggests progressive migration of Fe attached to the 316L side during welding. Furthermore, dimple formation was more prominent in specimens subjected to extended welding durations. The increasing appearance of these dimples with increasing welding time suggests ductile fracture behavior, which indicates improved joint integrity. Such an evolution in the fracture surface morphology reflects a positive correlation with the evolution of the interfacial strength, as shown in Fig. 2.

### Bonding development of the 316L/Ni/316L Interface

**Figure 7:**
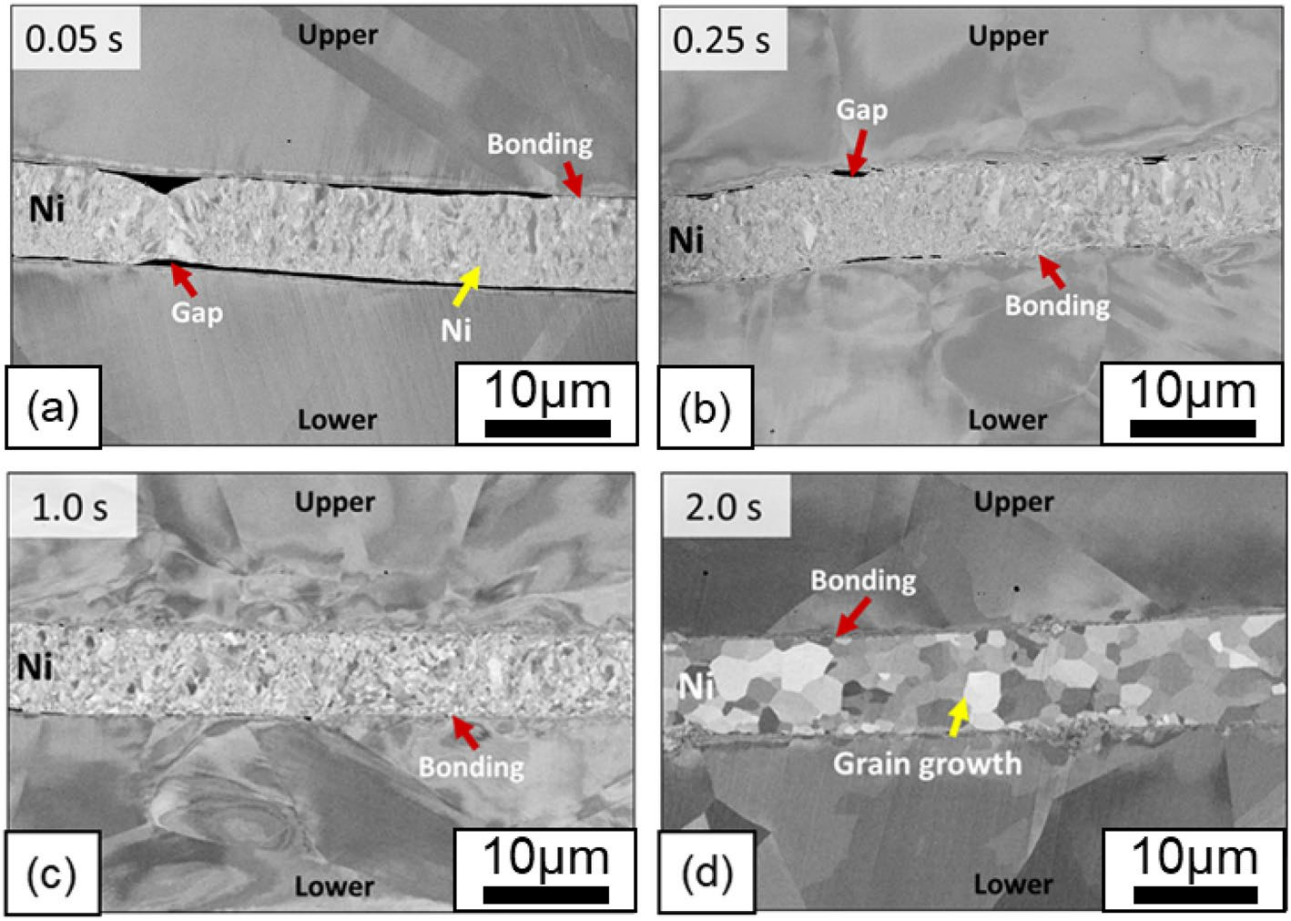
Interfacial microstructures of the 316L/Ni/316L interface after (**a**) 0.05 s, (**b**) 0.25 s, (**c**) 1.0 s, and (**d**) 2.0 s of welding.

Figure 7 shows the microstructure of the 316L/Ni/316L interface. Compared with the 316L/Fe/316L interface, only partial bonds were formed on the 316L/Ni and Ni/316L interfaces in the early stage of USW (0.05 s), as shown in Fig. 7a. This corresponds to a lower strength (less than 1000 N), as shown in Fig. 2. The dark areas between the 316L substrates and Ni interlayer are unbonded gaps. After 0.25 s of welding (Fig. 7b), the gaps disappeared significantly and were replaced by apparent bond formation, where the backscattered electron contrast suggested that deformation was evident at both the upper 316L/Ni and Ni/lower 316L interfaces.

As the welding time was increased to 1.0 s (Fig. 7c), bonded sections between the 316L/Ni and Ni/316L interfaces were gradually achieved, and only a few gaps remained at the upper 316L/Ni interface, indicating that the unbonded gaps were eliminated with welding time. Extending the welding time further to 2.0 s (Fig. 7d) resulted in the complete disappearance of the gaps at both interfaces, although some voids remained at the 316L/Ni interface, and only some of the voids remained at the lower 316L/Ni interface. Compared with the 316L/Fe/316L interface, the change in the thickness of the Ni interlayer was less pronounced than that of the Fe interlayer. This suggests that the Ni interlayer is not significantly deformed due to its higher hardness at elevated temperatures.

**Figure 8:**
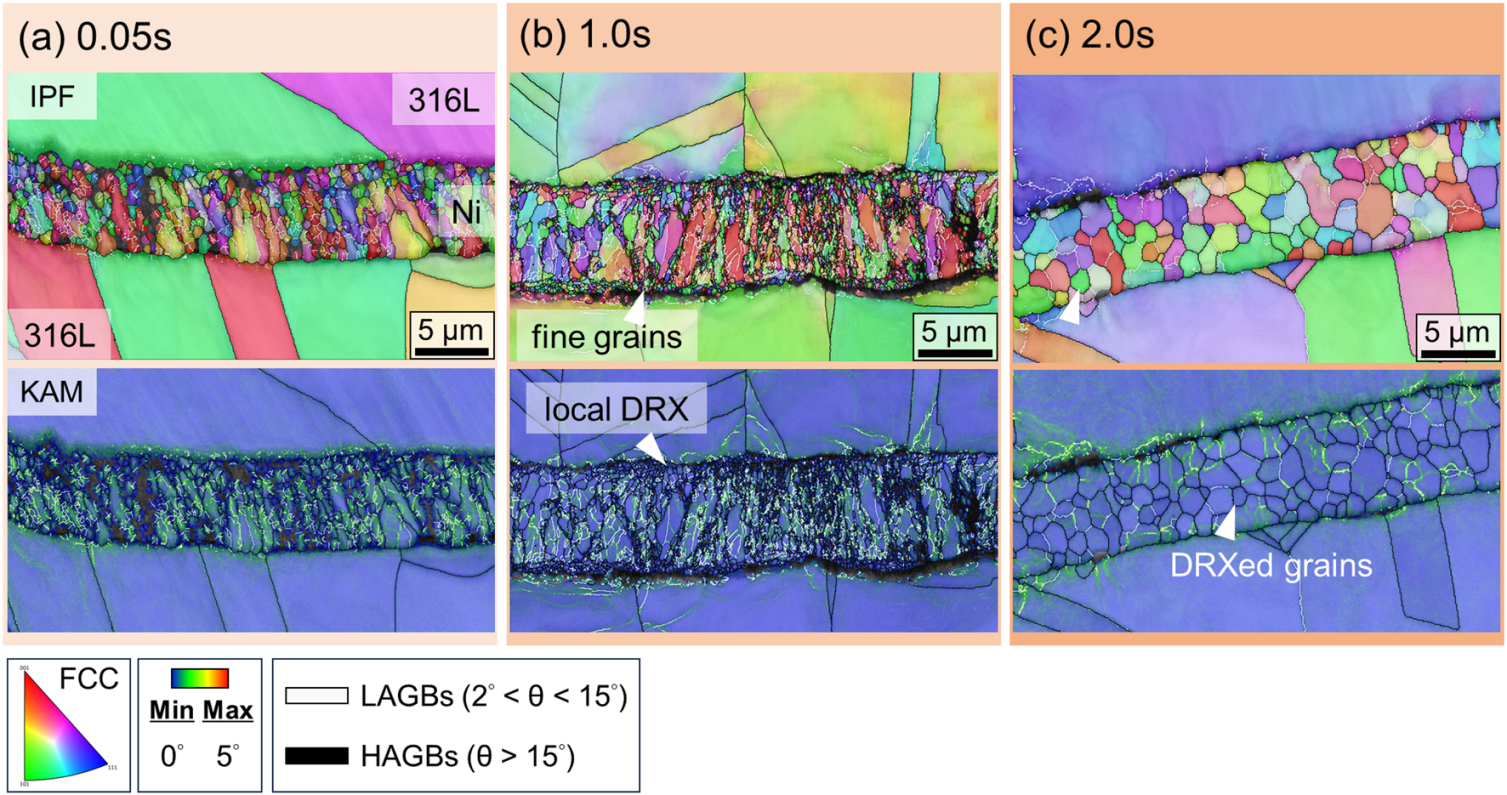
Time-dependent interfacial microstructures of the 316L/Ni/316L interface after (**a**) 0.05 s, (**b**) 1.0 s, and (**c**) 2.0 s of welding, characterized by EBSD.

Fig. 8 shows the EBSD results for the 316L/Ni/316L interface. At 0.05 s welding, as shown in Fig. 8a, the IPF map shows that the Ni grains in the interlayer exhibited a smaller change in the microstructure. The KAM map revealed that plastic strain was significant because of the cold-rolled microstructure. As the welding time increased to 1.0 s (Fig. 8b), the IPF and KAM maps showed that the formation of LAGBs became more evident, accompanied by more plastic strain in the Ni interlayer and 316L substrates. In other words, plastic deformation gradually propagates from the bonding interface along the vertical direction into the substrate. In addition, Ni grains with sizes of approximately one micron were locally formed near the interface with a necklace distribution in the Ni interlayer, suggesting the gradual development of DRX^49^ (see the arrow in the KAM map in Fig. 8b). As the welding time prolonged to 2.0 s (Fig. 8c), the IPF map revealed that the grains in the Ni interlayer became more equiaxed and were surrounded by HAGBs, whereas the LAGBs almost vanished. The KAM map reveals reduced plastic strain in the equiaxed grains, suggesting recrystallization.

**Figure 9:**
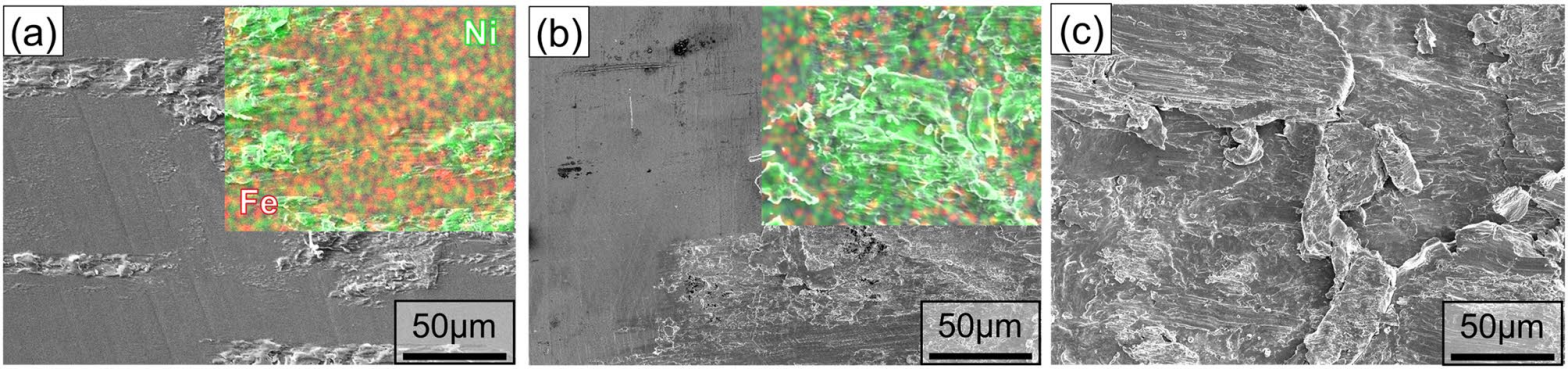
Fractured surfaces of 316L substrate after (**a**) 0.05 s, (**b**) 0.5 s, and (**c**) 1.5 s of welding time, characterized by SEM and EDS.

Figure 9 shows the fractured surfaces of the 316L substrate attached to the Ni foil after the lap shear test. The SEM–EDS overlaid images reveal that for a short welding duration (0.25 s), the attachment of the Ni interlayer on the 316L side was comparatively lower than that observed at the 316L/Fe/316L interface (Fig. 9a). The lower attachment was comparable to the cross-sectional observation, where the areas without the attachment of the Ni fragments corresponded to the gaps observed in Fig. 8a, suggesting bonding development at the early stage. After 0.5 s of welding, the fraction of Ni fragments attached to the 316L side increases slightly (Fig. 9b). This can be attributed to the higher hardness of Ni compared to that of Fe. As the welding time increased to 1.5 s, the fractured surface in Fig. 9c exhibited a brittle fracture, corresponding to an interfacial strength of less than 2000 N. The Ni interlayer required a longer welding duration to evolve bonding with a greater temperature elevation than the Fe interlayer.

### Evolution of interfacial temperature with or without interlayers

**Figure 10:**
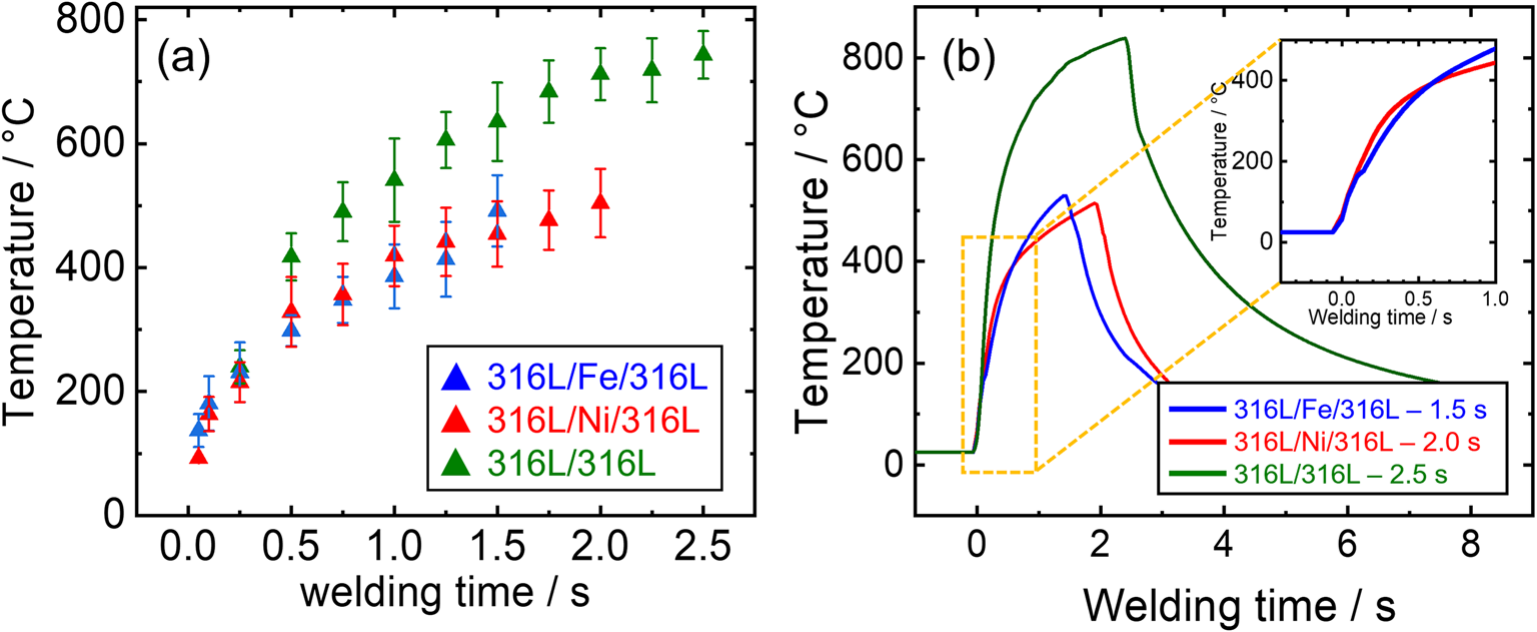
(**a**) Interfacial temperature evolution plotted as a function of welding time with and without interlayer (**b**) representative temperature profiles at long weld durations.

Fig. 10a quantitatively depicts the peak temperatures of the 316L/316L joints with and without interlayers. The 316L/316L interface exhibited a pronounced and rapid temperature rise, reaching 400 °C after 0.5 s and 500 °C after 1.0 s welding. Conversely, the Fe and Ni interlayers (the 316L/Fe/316L and 316L/Ni/316L interfaces) demonstrated a moderate temperature increase at a slower rate than the Al interlayer, which could be attributed to three-body friction. The peak temperatures of the Fe interlayer reached 450 °C after 1.5 s welding. Conversely, the Ni interlayer reached 450 °C after a longer welding duration of 2.0 s.

The 316L/316L interface presented a gradual temperature increase, achieving 520 °C at 1.0 s and culminating at 750 °C after 2.5 s welding, revealing a more pronounced increment than using Fe or Ni interlayer. This difference can be attributed to energy dissipation in the sliding behavior. The frictional heat generated at the 316L/316L interface was higher under two-body sliding than that in the 316L/Fe/316L and 316L/Ni/316L samples under three-body sliding conditions. Because the energy applied by the equipment can be dissipated in the form of elastic deformation^50^ or heat sinking^51^, this different sliding behavior could lead to a difference in the elevation of the interfacial temperatures.

Figure 10b shows the long-term thermal cycles for the 316L/316L, 316L/Ni/316L, and 316L/Fe/316L interfaces, illustrating the temperature changes during USW. All cases showed a steady and rapid increase during USW. Upon completion of USW, cooling was rapid owing to contact with the horn and anvil, where different cooling rates could be attributed to different temperature gradients. Regarding response time for K-type thermocouples, it is reported that thin wires (0.5 mm in diameter or less) could reveal a rapid response time (0.1–0.5 s)^52^. Thus, thin K-type thermocouple wires (0.127 mm in diameter) were employed because of their quick response times for recording rapid changes in USW. Despite the rapid response, there can be discrepancies in the temperature measurements owing to the position of the thermocouple and the effects of internal heat conduction of thermocouple wire, especially with thicker wires. Note that the temperature values at a very early stage (before 0.1 s) might be less reliable, as shown in the 316L/Fe/316L interface showing a two-stage temperature increase (indicated by the red arrows in the inset of Fig. 10b).

Moreover, measured temperature values were aligned with microstructural changes observed in longer-duration welds, such as DRX and grain growth in Ni and Fe interlayers. These are reported metallurgical phenomena in the measured temperature ranges^4^. Despite the inevitable deviation between the measured and actual temperatures, the measured temperatures were consistent with the microstructural changes observed in the longer-duration welds, such as DRX at the 316L/Fe/316L (Fig. 5c) interface and grain growth at the 316L/Ni/316L interface (Fig. 8c).

## Discussions

### Influence of peak temperature on interfacial strength

**Figure 11:**
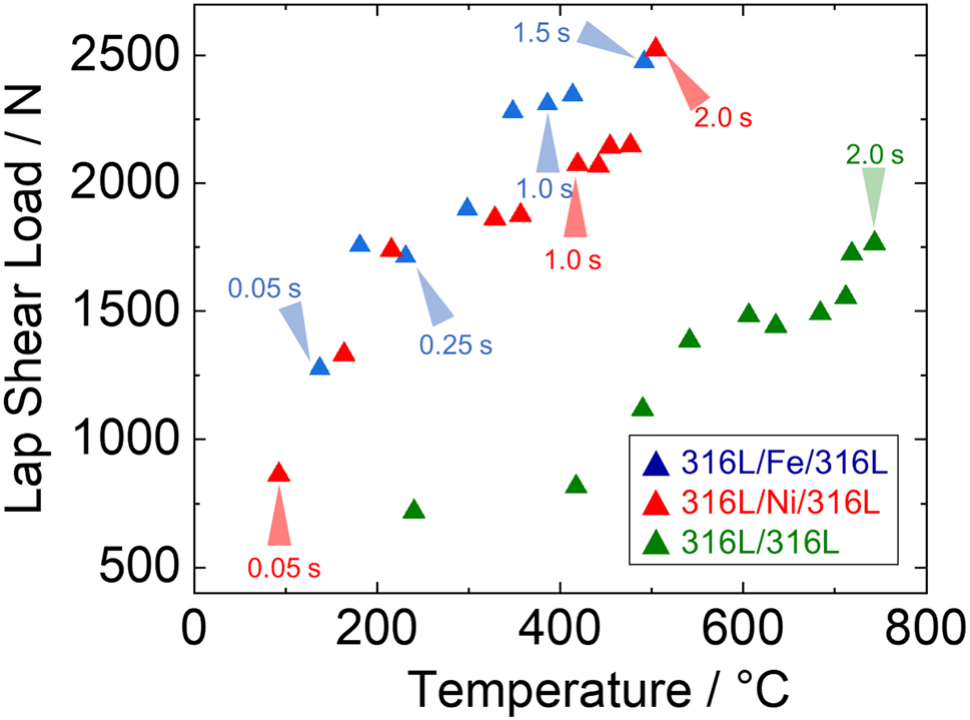
Relationships between interfacial strength and interfacial temperature.

Figure 11 shows the relationship between the interface temperature and welding time and between the lap shear strength and the interface temperature. The 316L/Fe/316L, 316L/Ni/316L, and 316L/316L interfaces exhibit more significant increases than the 316L/316L interface. Regarding this difference in temperature increase during USW, it is considered that at the early stage of USW (interfacial temperature < 200 °C), sliding friction caused a rapid bond production between substrates by collapsing asperities under sliding contact. During this process, the Fe and Ni interlayers underwent a greater extent of plastic deformation.

As the temperature increased further, the decrease in hardness led to a difference (referring to 1.0 s in the 316L/Fe/316L and 316L/Ni/316L) in strength, where Ni (144 HV_0.05_ at room temperature) possessed a higher hardness and slower hardness decrease than Fe (84 HV_0.05_ at room temperature). Interfacial strength peaked at 2500 N as temperature increased to about 500 °C (1.5 s in the 316L/Fe/316L interface and 2.0 s in the 316L/Ni/316L interface). Comparatively, the 316L/316L interface exhibited a slower increase compared with the above two cases, which could be attributed to the higher resistance to plastic deformation, leading to a lower strength even with an interfacial temperature near 800 °C (after 2.0 s of welding).

**Figure 12:**
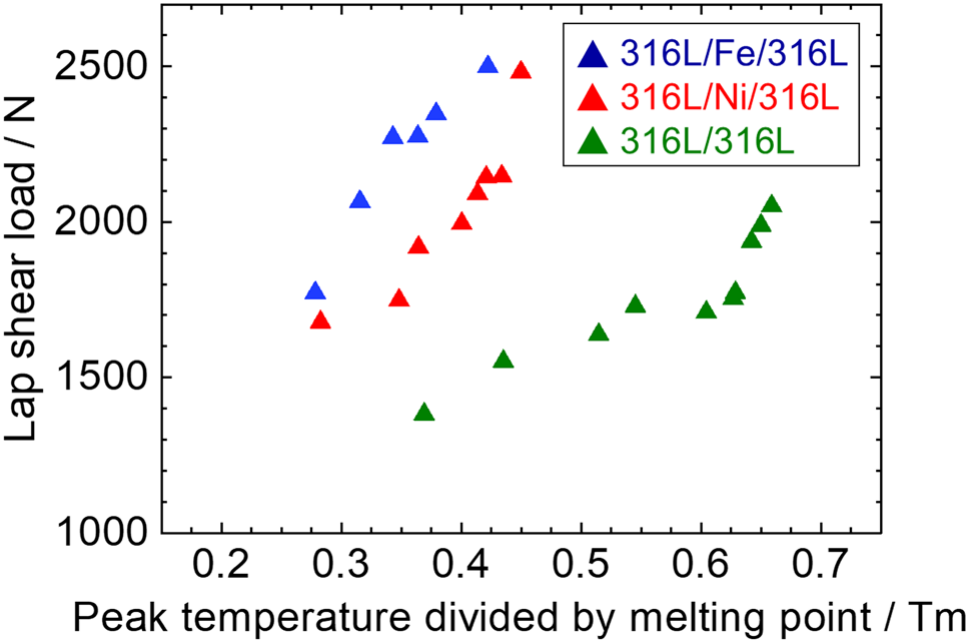
Relationship between interfacial strength and peak temperature divided by melting point.

Figure 12 shows interfacial strength evolution plotted against peak interfacial temperatures for three cases, where peak interfacial temperatures (the 316L/316L, 316L/Fe/316L, and 316L/Ni/316L interfaces) were homogenized by melting points of 316L, Fe, and Ni, respectively (the melting point of Fe and Ni are 660, 1538, and 1455 °C). For joint with interlayer, it is evident that peak temperatures interfaces reached 0.4–0.5 T_m_ of pure Fe and Ni. Briefly, the 316L/Fe/316L interface exhibited a steady increase in strength as the peak temperature exceeded 0.3 T_m_ and reached the highest strength under 0.41 T_m_. In contrast, the 316L/Ni/316L interface exhibited a slower increase, with a peak temperature of 0.3–0.35 T_m_. As the peak temperature increased above 0.35 T_m_, the interfacial strength increased faster and peaked above 2500 N at 0.44 T_m_. In comparison, the 316L/316L interface exhibits a significantly slower increase in strength when the peak temperature is < 0.6 T_m_. As the interfacial temperature increased above 0.6 T_m_, the interfacial strength rapidly increased and peaked at a maximum value of approximately 0.67 T_m_.

The relationship between temperature and hardness is well-defined; an increase in temperature is recognized to impact the decrease in hardness^48^ significantly. In other words, the hardness decreases slowly at low temperatures until the temperature reaches the transition point. As temperature exceeds this point, hardness decreases drastically, where the transition point is possessed at 0.4–0.5 T_m_ for pure metals, while this value could be increased in alloys, such as stainless steels. The hardness of the transition points at elevated temperatures was reported to be 0.4 T_m_ for pure Fe and 0.42 T_m_ for pure Ni, which is in good agreement.

In addition, the strength of the 316L/316L interface increases considerably as the temperature increases above 0.62 T_m_. This could be attributed to the fact that^53^ 316L SS revealed a lower reduction in hardness and yielding strength when the temperature was no more than 800 °C (0.64 T_m_), where a yielding strength of 140 MPa at 750 °C and decreased to 110 MPa at 825 °C, and a significant reduction in yielding strength to 60 MPa could be obtained above 900 °C (0.7 T_m_). This insignificant reduction in hardness at elevated temperatures explains why the 316L/316L interface exhibited a slight increase in the interfacial strength when the temperature was below 0.6 T_m_. The higher hardness resulting from sliding and nanograin formation could reduce the deformability for further plastic strain, leading to defect formation and limiting the strength increment.

Furthermore, considering the effect of the clamping force (i.e., compression), yielding at elevated temperatures could be the main factor affecting bonding. Because a clamping force (3000 N) was applied to a weld area of 10 × 10 mm, the average clamping stress was first evaluated as 30 MPa and uniformly applied to the surface. However, it has been reported that the sharp edge of the knurled surface of the horn has a concentrating effect on the clamping forces on the workpieces^54^, resulting in the concentration of a considerable magnitude of clamping stress at the tips of the horn teeth. Combining these conditions, the stress concentration on the knurled surface, accompanied by a rapidly increasing temperature, might be responsible for the yielding of the Fe and Ni interlayers but has a negligible effect on the 316L substrate, thereby leading to a more rapid bonding development at a short welding duration.

### Effect of deformation behavior at elevated temperature on interfacial integrity

**Figure 13:**
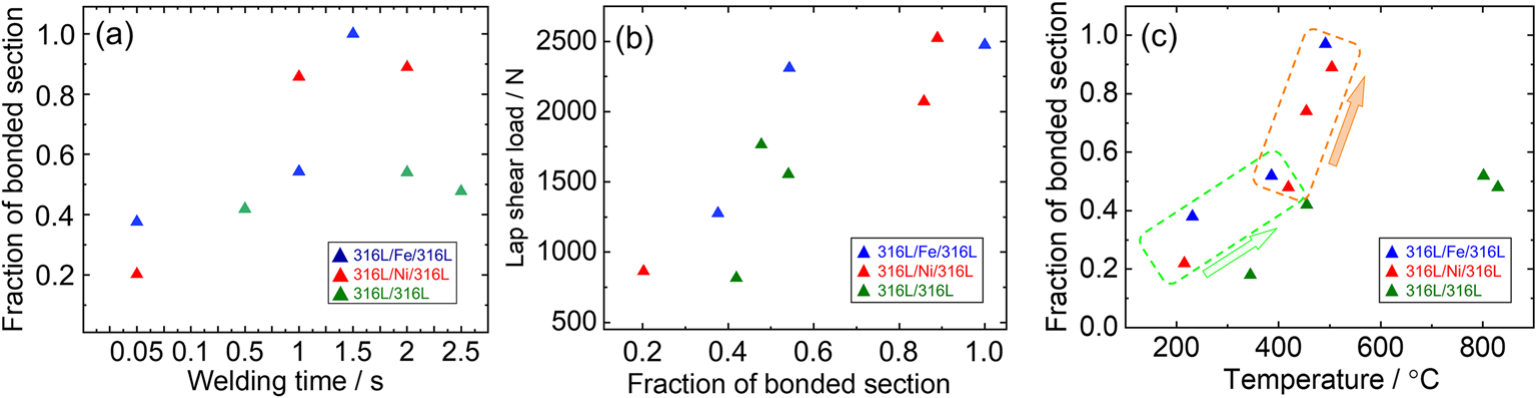
(**a**) Relationship between bonded section and welding time. (**b**) Strength evolution plotted as a function of bonded section. (**c**) Relationship between bonded section and interfacial temperature.

Based on the above, it is considered that an elevated temperature has a significant influence on interface deformability and bonding integrity. Fig. 13a shows the bonded section plotted against the welding time, where the bonded section was measured from multiple SEM images of the three cases. For the 316L/Fe/316L and 316L/Ni/316L interfaces, the bonded section increased rapidly to above 0.8 within 2.0 s of welding. Conversely, the 316L/316L interface only slightly increased the bonded section even after 2.5 s welding. Figure 13b shows a positive relationship between the bonded sections, indicating that the evolution of the interfacial strength was similar for the three cases.

Regarding bond formation, Fig. 13c shows the evolution of the bonded section with the interfacial temperature. The 316L/Fe/316L and 316L/Ni/316L interfaces revealed a transition as temperature increased above 400 °C, suggesting the hardness drop in the interlayer and its effect on bonding integrity. By contrast, the 316L/316L interface exhibited a slower increase in the bonded section, attributed to its higher resistance to deformation at elevated temperatures.

Because DRX is a significant phenomenon during USW^55^, to understand the effect of elevated temperatures and deformation on the microstructures of the interlayers, the Zener–Hollomon parameter (Z)^56^ was utilized to evaluate the degree of dynamic recrystallization based on the measured peak temperature, which is given by Eq. (1).

1 $$Z = \in \,\,\exp \,(\frac{Q}{RT})$$

where ϵ is the strain rate, Q is the activation energy for deformation (self-diffusion for recrystallization), R is the gas constant, and T is Kelvin temperature. As for strain rate, a constant value of 10^3^ s^-1^ was given based on previous works^57^. Based on the given activation energies for self-diffusion in pure Fe^58^ and Ni^59^, the Z parameters were estimated and are summarized in Table 2.

**Table 2:** Summary of Z parameters on DRXed specimens.

Interlayers	Welding time/s	Peak temp./°C	DRXed grain size/µm	Q (kJ/mol)	Z	lnZ
Fe	0.25	298.3	0.31	248	1.8 × 10^28^	65.1
Fe	1.0	385.3	0.47	248	7.9 × 10^24^	57.3
Ni	1.0	418.9	0.81	187	1.3 × 10^17^	39.4
Ni	2.0	504.1	1.64	187	3.8 × 10^15^	35.8

R: 8.314 J/mol‧K.

As for DRX reported in USW, the ln Z values range from 30–60^9,55,60^, which is comparable to this study but larger than those reported in other plastic processing methods (e.g., 20–40 ln Z in friction stir processing^61^). For high Z values, grain nucleation via discontinuous DRX (DDRX) could be more dominant than grain growth because a lower temperature is beneficial for grain nucleation^62^, corresponding to the refined grains observed in the Fe interlayer after 1.0 s of welding. Comparatively, as the temperature increases, ln Z decreases correspondingly, whereas lower ln Z values suggest a higher driving force for grain growth because of higher temperatures^63^. These could be aligned with the larger Ni grains via DRX after 2.0 s of welding. In addition, the lower activation energy for the diffusion of Ni could result in a more apparent growth of Ni than that of Fe. With larger DRX grains, the Ni interlayer exhibited greater compatibility with plastic deformation near the interface, leading to a rapid increase in the bonding section.

## Conclusion

This study investigated the effect of interfacial deformability on bond formation and the corresponding interfacial strength and found that higher deformability near the interface is important for bonding integrity, which can be summarized as follows:

1. The 316L/316L interface exhibited a slight increase after 2.5 s welding, where continuous sliding at the interface caused internal crack formation, thereby limiting strength evolution. This could be attributed to the higher resistance of plastic deformation of 316L when the interfacial temperature was less than 0.6 T_m_.
2. The 316L/Fe/316L and 316L/Ni/316L interfaces positively affected the interfacial strength (peak load of 2500 N) within a short welding duration. The relationship between the interfacial strength and peak temperature showed that a significant drop in hardness at 0.4 T_m_ is crucial for obtaining extensive bond formation.
3. Significant plastic flow was produced at the 316L/Fe/316L and 316L/Ni/316L interfaces by DRX, where grains were refined in the Fe interlayer owing to the shorter weld duration, and grain growth was predominant in the Ni interlayer owing to its lower activation energy. The Fe and Ni interlayers enhance the bonding integrity because of their excellent accommodation of plasticity without damaging the 316L substrate.

### Supplementary Information

Supplementary Figures.

## Data Availability

The datasets used and/or analysed during the current study is available from the corresponding author upon reasonably request.
