# Supplementary material for: Enhanced strength of ultrasonically-welded austenitic stainless steels joints by introducing dynamic recrystallization of interlayers
Source: Sci Rep. 2024 Aug 2;14:17925. doi: 10.1038/s41598-024-66205-8 (PMC11297197; doi:10.1038/s41598-024-66205-8)
Supplement: Supplementary file 1 — Supplementary Figures. [file 41598_2024_66205_MOESM1_ESM.docx]

**Supplementary material**

**
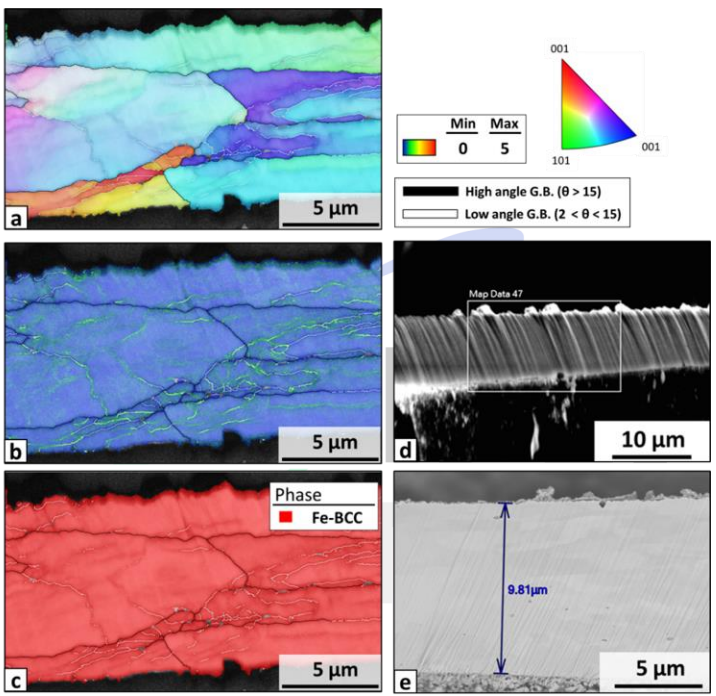
**

**Figure S1** SEM and EBSD results of Fe foils before USW, including (a) IPF map (b) KAM map (c) phase map (d-e) SEM image

**
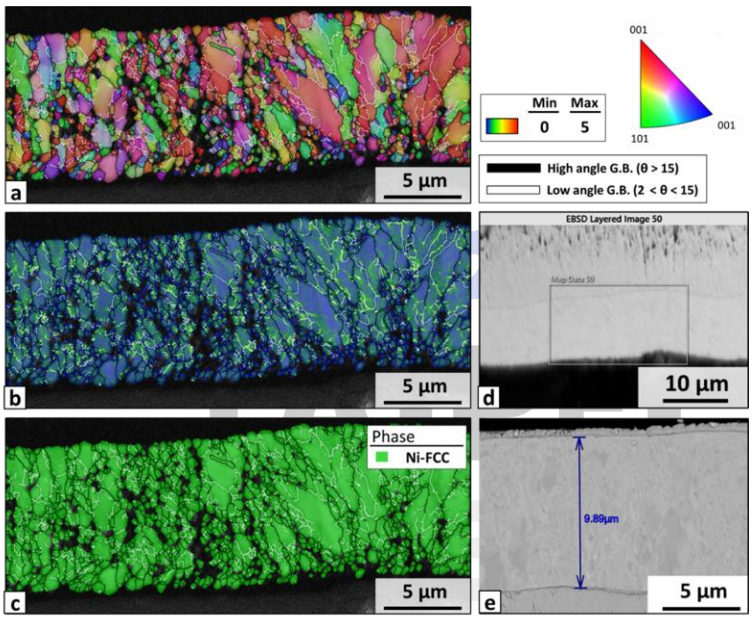
**

**Figure S2** SEM and EBSD results of Ni foils before USW, including (a) IPF map (b) KAM map (c) phase map (d-e) SEM image

**
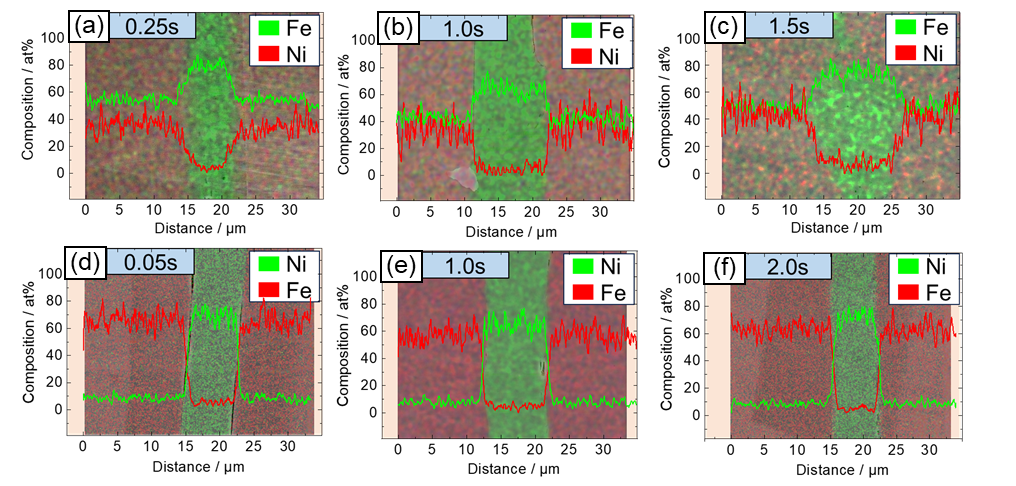
**

**Figure S3** SEM-EDS maps incorporated with line scan profiles of (a-c) 316L/Fe/316L interface and (d-f) 316L/Ni/316L interface.
